# The deployment of mobile diagnostic laboratories for Ebola virus disease diagnostics in Sierra Leone and Guinea

**DOI:** 10.4102/ajlm.v10i1.1414

**Published:** 2021-10-22

**Authors:** Lance D. Presser, Jeanette Coffin, Lamine Koivogui, Allan Campbell, Julian Campbell, Fatmata Barrie, Jone Ngobeh, Zein Souma, Samuel Sorie, Doris Harding, Alimou Camara, Pepe Tohonamou, Basala Traore, Frank A. Hamill, Joe Bogan, Sharon Altmann, Casey Ross, Jay Mansheim, Robert Hegerty, Scott Poynter, Scott Shearrer, Carmen Asbun, Brendan Karlstrand, Phil Davis, Jane Alam, David Roberts, Paul D. Stamper, Jean Ndjomou, Nadia Wauquier, Mohamed Koroma, Alhaji Munu, Jason McClintock, Mar Mar, True Burns, Stephen Krcha

**Affiliations:** 1 Global Engagement Program, MRIGlobal, Gaithersburg, Maryland, United States; 2 Centre de Recherche et de Formation en Infectiologie de Guinée, Université Gamal Abdel Nasser de Conakry, Conakry, Guinea; 3 Central Public Health Reference Laboratory, Freetown, Sierra Leone; 4 Institut National de Santé Publique, Conakry, Guinea

**Keywords:** Ebola, Ebola virus, Sierra Leone, Guinea, diagnostics, laboratory capacity, service expansion, epidemic, outbreak, outbreak response, West Africa, mobile diagnostic laboratories

## Abstract

**Background:**

Ebola virus emerged in West Africa in December 2013. The ease of mobility, porous borders, and lack of public health infrastructure led to the largest Ebola virus disease (EVD) outbreak to date.

**Intervention:**

The 2013 EVD outbreak signalled the need for laboratory diagnostic capabilities in areas without strong public health systems. As part of the United States’ Department of Defense response, MRIGlobal was contracted to design, fabricate, equip, deploy, and operate two mobile diagnostic laboratories (MDLs). The first laboratory analysed blood samples from patients in an adjacent Ebola Treatment Centre (ETC) and buccal swabs from the deceased in the community in Moyamba, Sierra Leone. The second laboratory was deployed to support an ETC in Conakry, Guinea. The Department of Defense provided real-time quantitative reverse transcription polymerase chain reaction assays that were deployed and validated on-site.

**Lessons Learnt:**

Prompt and accurate molecular diagnostics reduced sample turn-around times from over 24 h to under 4 h. Experienced laboratory staff tested up to 110 samples per day and on-site engineering proved necessary for MDL setup and operation. As the Ebola response slowed, the sustainment of the MDLs’ operations was prioritised, including staff training and the transition of the MDLs to local governments. Training programmes for local staff were prepared in Sierra Leone and Guinea.

**Recommendations:**

The MRIGlobal MDL team significantly contributed to establishing increased laboratory capacity during the EVD outbreak in West Africa. Using the MDLs for molecular diagnosis is highly recommended until more sustainable solutions can be provided.

## Background

In March 2014, the World Health Organization was notified regarding a cluster of disease in Guinea characterised by fever, severe diarrhoea, vomiting, and high fatality rate. Eventually, the disease was identified as a novel strain of the Ebola virus.^1^ Further investigation suggested the first fatality of the outbreak had occurred in December 2013 in Guinea.^1^ When the outbreak was declared over, 28 616 Ebola virus disease (EVD) cases including 11 310 total deaths had been reported.^2^ The unprecedented scale of this EVD outbreak resulted in sustained human-to-human transmission, the consequences of which are still being elucidated.

Ebola virus is a biosafety level 4 (BSL-4) agent. Specimen inactivation should be performed in at least a BSL-3 laboratory, after which routine diagnostic specimen testing can be performed in a BSL-2 laboratory. When the outbreak began, the closest BSL-3 laboratory was in Nigeria and was being used for tuberculosis diagnostics. It was not capable of EVD testing, in that it lacked polymerase chain reaction (PCR) machines and had no validated assay and reagents. Similarly, the closest BSL-4 laboratory was situated in Gabon, 3000 km away from Freetown, and unable to assist with EVD diagnostics timeously. None of the West African countries hit hardest by the EVD outbreak (Guinea, Sierra Leone, or Liberia) had adequate EVD diagnostic facilities; this necessitated the development of mobile diagnostic laboratory (MDL) units, and improvement of other laboratory capacities in the region to help control the outbreak.

The United States Department of Defense initiated the Cooperative Biological Engagement Program in West Africa through the Defense Threat Reduction Agency to contain the biological agent (Ebola virus), enhance biosafety and biosecurity, and strengthen the region’s ability to detect, diagnose, and report public health emergencies of international concern to the World Health Organization.^3^ MRIGlobal was awarded the contract to design, assemble, equip, and deploy rapid response MDLs for molecular detection of Ebola virus in patient samples.

At the invitation of the Sierra Leonean and Guinean governments, as well as the Department of Defense, the non-profit organisation MRIGlobal designed, built, delivered, and operated MDLs during the EVD outbreak starting in December 2014. By 2016, MRIGlobal had shifted its focus from emergency response to a smooth transition of management, which included staff training and support to the Central Public Health Reference Laboratory in Sierra Leone and the National Institute of Public Health in Guinea. We describe here the deployment of the MDLs for the EVD outbreak response and discuss the successes and challenges experienced.

## Description of the intervention

### Ethical considerations

The EVD outbreak response was declared a public health emergency of international concern by the World Health Organization on 08 August 2014. The standard operating procedures used for diagnostic testing were approved by the World Health Organization, the Department of Defense, and the Ministry of Health in Guinea and Sierra Leone. Diagnostic results were released as quickly as possible following specimen analyses. Neither MRIGlobal nor the Department of Defense retained any samples as they were either destroyed or turned over to the host country.

### Mobile diagnostic laboratory design

From an engineering standpoint, the project’s goal was to build a mobile, self-sustained, self-contained (safe) laboratory ready for delivery in less than six weeks. MRIGlobal engineers selected 20-foot (~6.1 m) intermodal containers because they provide a rugged, watertight, customisable shell that can be easily transported. MRIGlobal has over 15 years of experience in designing, building, maintaining, and deploying similar containerised laboratories around the world. MRIGlobal engineers had to consider customisation such as including surfaces that were easy to decontaminate and providing attachment and stabilisation points for all pieces of equipment within the labs, including lighting, heating, ventilation, and air conditioning systems.

**FIGURE 1:**
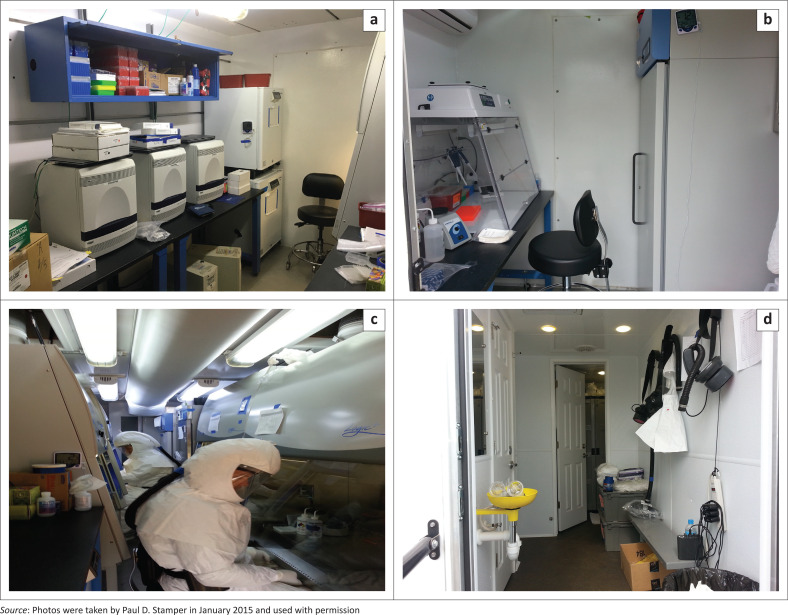
MRIGlobal mobile diagnostic laboratory that was deployed in response to the West Africa Ebola virus outbreak. (a) Interior of polymerase chain reaction laboratory unit. (b) Interior of master mix laboratory. (c) Interior of inactivation and extraction laboratory unit. (d) Interior of locker room.

MRIGlobal’s engineers and scientists worked together to design the laboratory to ensure safe handling and testing of samples and the accommodation of equipment required for operations in both countries. The laboratories were to include three separate areas – sample inactivation and extraction, reagent preparation, and quantitative reverse-transcription polymerase chain reaction (qRT-PCR) areas inside the two laboratory containers. The first container housed the sample inactivation and extraction area where infectious samples would be processed and inactivated inside of a Class II Type B2 biological safety cabinet (BSC). Four BSCs were placed in the inactivation laboratory to handle the anticipated sample volume. After delivery, modifications to the container were necessary to allow for sample pass-through between the two containers and to properly exhaust the BSCs (Figure 1c).

A partition was built inside the second laboratory container to make two separate work areas for the reagent preparation area and the qRT-PCR area (Figure 1a and 1b). Due to concerns about possible contamination in the reagent preparation area, additional air handling equipment was used to provide positive pressure to the reagent preparation area, thus ensuring it would remain clean.

Office space, supplies store, personal protective equipment (PPE) locker rooms, restroom, shower room, and a tool warehouse were also deemed necessary. A standard container was used to provide both office space and supply storage. MRIGlobal worked with a mobile restroom manufacturer to design a mobile trailer that would provide restroom and shower facilities as well as a locker room (Figure 1d). An additional small container was used as the tool warehouse and office space for the on-site engineer.

### Mobile diagnostic laboratory transportation and installation

**FIGURE 2:**
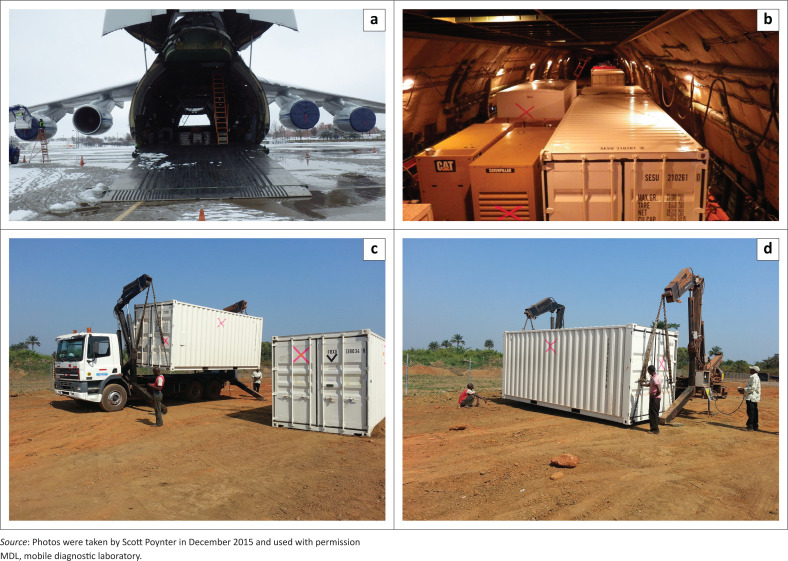
Transport of MRIGlobal MDL from the United States to Guinea and Sierra Leone. (a) Antonov An-124 aircraft used to transport the MRIGlobal MDL from Kansas City, Missouri, United States, to Guinea and Sierra Leone sites. (b) Interior of Antonov An-124 aircraft, loaded with MDL units. (c) Truck unloading MDL units in Moyamba, Sierra Leone. (d) Hydraulic system used to unload MDL units in Moyamba, Sierra Leone.

MRIGlobal was responsible for arranging transportation of these laboratories to Guinea and Sierra Leone. To best satisfy the schedule requirements, air cargo was used. The Aviastar-SP Antonov An-124 Ruslan (Figure 2) was the only aeroplane option due to some issues including the MDL size and cargo weight and the runway length in Guinea and Sierra Leone.

Once the equipment arrived at the sites, laboratory equipment was unpacked. The BSCs were certified by the on-site engineers and the electrical lines were run. However, the engineering team was faced with technical challenges relating to the electrical power supply, safe water and sewer connections, laundry facilities, biohazard waste disposal, and internet connectivity.

The MDLs were built with the United States electrical standards of 120 volts and 60 hertz. These electrical standards are not shared in Guinea or Sierra Leone and this resulted in difficulties replacing or servicing equipment. Also, there was the issue of an unstable power supply. To address the unstable power supply, two diesel generators were purchased, and fuel contracts were established. Each generator could provide light for the entire laboratory system on its own. However, the generators were significantly larger than necessary, resulting in reduced efficiency of the generators and increased fuel expenses. An automatic transfer switch was used to continuously monitor the power produced by the generator.

To address water availability and to provide a sewer connection and a laundry facility, a small container was used to house the water equipment for the laboratory, including the two safety showers which were located directly outside of the laboratory exit. The container housed a water pump, pressure bladder tanks, and the laundry facility. The system was designed so it could accept water from a supplied water line or a tank stored on top of the water container, depending on what was available when the laboratory reached its final destination. The restroom and shower trailers were built with black water storage tanks but were also capable of being connected to a septic or sewer system.

To ensure proper disposal of biohazard waste, a medical incinerator (Elastec Mediburn, Carmi, Illinois, United States) capable of temperatures above 1000 °C was installed. This ensured all infectious and pathological waste generated by the laboratory was safely disposed of.

The environment in Guinea and Sierra Leone also presented challenges. The MDLs were consistently exposed to high temperatures, high humidity, and heavy rains, which resulted in the rapid decomposition of many elements of the MDLs. These were addressed using guidelines and assessment tools provided in the ‘Report on the Status of Emerging and Dangerous Pathogen Laboratory Network BSL-3 in Select Countries in the African Region’.^4^ When possible, repairs and parts were sourced locally. When local repairs or parts were not available, they were included in quarterly laboratory supply shipments that originated in the United States.

### Staff composition and worksite layout

**FIGURE 3:**
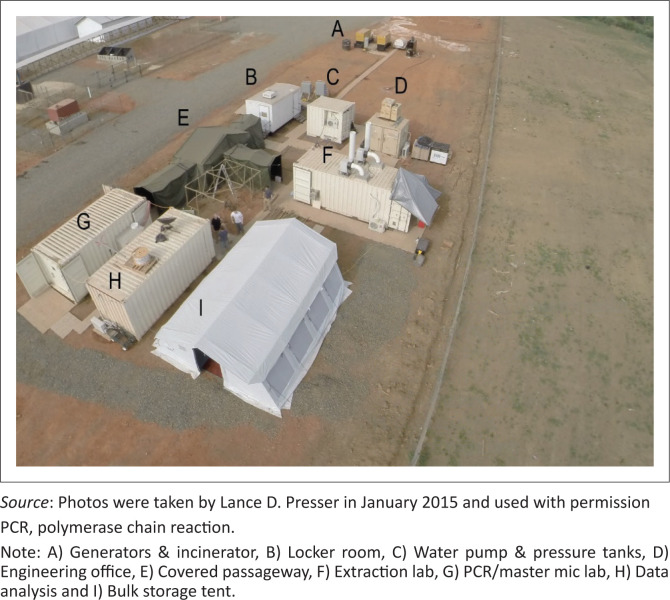
Worksite layout for the MRIGlobal mobile diagnostic laboratory, Moyamba, Sierra Leone. Each laboratory unit is labelled appropriately.

In Sierra Leone, the MDL arrived on 18 December 2014 and sample testing started on 12 January 2015 (25 days). In Guinea, the time between the arrival of the MDL arrival and the start of sample testing was shortened to 13 days (21 April 2015 to 04 May 2015). The composition of the MDL team was a rotation of four scientists, two engineers, and multiple drivers at each site. The initial site layout for both Sierra Leone and Guinea is shown in Figure 3.

#### Specimen collection

Blood and swab specimens were delivered to the MDL sites in Conakry, Guinea and Moyamba, Sierra Leone, primarily by motorbike couriers or from the adjacent Ebola Treatment Centre. When receiving the specimens, the staff wore coveralls, sleeves, and double gloves. PPE was stored and donned in the locker room unit (Figure 1d). The surface of the specimen bucket and the sample packaging bag were disinfected by spraying with 0.5% hypochlorite solution.

#### Specimen inactivation and nucleic acid extraction

The MRIGlobal Ebola response team inactivated samples in the inactivation and extraction laboratory unit (Figure 1c) wearing appropriate PPE. The PPE included coveralls, sleeves, triple gloves, and a powered air-purifying respirator system. The sample transport container was disinfected with 0.5% hypochlorite solution and opened within the BSC. For samples requiring malaria testing, the Malaria Ag P.F. test (SD Bioline, Chicago, Illinois, United States) was performed. Whole blood or plasma sample inactivation was performed using Trizol Liquid Sample (ThermoFisher, Waltham, Massachusetts, United States).

Following inactivation, sample RNA was extracted using the EZ1 Virus Mini Kit version 2.0 in the EZ1 Advanced XL hardware platform (Qiagen, Hilden, Germany). Extracted RNA from samples was immediately sent for qRT-PCR amplification or stored at –20 °C.

#### Quantitative reverse transcription polymerase chain reaction assays

The qRT-PCR assays were performed using the Ebola Zaire TaqMan and Ebola Zaire TaqMan-MGB assays provided by the Department of Defense and Joint Program Manager-Medical Countermeasure Systems Critical Reagents Program (Figure 1a). The assays were authorised for use on the 7500 Fast Dx Real-Time PCR Instrument (Applied Biosystems, Foster City, California, United States).^5^ The multiplex PCR steps were programmed as follows: Stage 1 – 15 min at 50 °C, Stage 2 – 5 min at 95 °C, Stage 3 – 1 s at 95 °C, 26 s at 60 °C, Stage 4 – 30 s at 40 °C.^5^

### Specimen storage

The MDL was equipped with –20 °C freezers. As a result, only short-term (< 14 days) storage of patient samples was maintained. The specimens were well packed and the surface disinfected by 0.5% hypochlorite solution before storage. The MDL sites were guarded round the clock and all freezers and containers were locked.

### Test result release

Samples from patients were assigned an individual, unique identification number by the emergency operations, established by each country’s Ministry of Health. When a sample was collected, the clinician was asked to complete a ‘viral haemorrhagic fever case investigation form’. The sample and the investigation form were marked with the case identification number and patient name and promptly transported to the laboratory. The case identification number provided a unique number for tracking the specimen from the patient, and the results of the specimen. The information in the test report included the case identification number, the Ct value determined by qRT-PCR, and the confirmed result (Yes, No, or Suspect). According to the agreement with the health authorities, the MRIGlobal Ebola response team did not contact hospitals directly. Instead, MRIGlobal submitted the test report to the World Health Organization and a local emergency operations centre, which delivered consistent and timely results to each hospital and treatment centre.

#### Transfer of ownership

The United States government supported the transfer of ownership of the supplies, materials, and equipment to the Ministry of Health of Guinea on 23 September 2016 and to the Ministry of Health of Sierra Leone on 16 March 2017. Personnel from each recipient country were trained before the transfer of ownership to allow for long-term independent maintenance of the improved diagnostic capabilities. In preparation for the transfer of ownership to the Ministry of Health of Guinea, MRIGlobal developed and provided a series of trainings, each followed by a test and evaluation. Training included tabletop exercise and a field operational demonstration. The tabletop exercise was designed to engage ministries of health and local and international partners in a collaborative effort to define how these new facilities and capabilities can best be merged into the existing national laboratory response systems and to capture stakeholder recommendations for improving long-term sustainability. The field training exercise was observed by evaluators and referees to gauge the capability of the newly trained staff in performing essential laboratory functions safely and effectively. The transfer of laboratory capacity to Sierra Leone and Guinea is aimed at helping both countries fulfil the ‘Core Capacity Requirements for Surveillance and Response’ as outlined in the International Health Regulations 2005 Annex 1.^6^

#### Other partner capabilities

Many other international partners played a role in the EVD response in West Africa, and there were numerous types of laboratories and laboratory diagnostics deployed. The Dutch government deployed three MDL units, the Chinese government deployed one MDL unit and built a BSL-3 laboratory and hospital outside of Freetown, Sierra Leone, and the United States Centers for Disease Control and Prevention established a field laboratory in Bo, Sierra Leone, alongside a host of other partner activities.^7,8,9,10^ The two MDL laboratories that were deployed by MRIGlobal tested 18 624 total samples without a safety incident. Having adequate laboratory capacity, provided almost entirely by international partners, was key to meeting sample turn-around time criteria, proper diagnosis, contact tracing, and ultimately containment of the EVD outbreak.

## Lessons learnt

The challenge assigned to MRIGlobal by the Department of Defense was to quickly deploy safe and effective laboratory diagnostic capabilities to Sierra Leone and Guinea to address the EVD outbreak. Numerous international partners were or became involved in Guinea and Sierra Leone, including but not limited to the United States Centers for Disease Control and Prevention, the Chinese Centre for Disease Control and Prevention, Médecins Sans Frontières International, Public Health England, World Health Organization, Partners in Health, the Dutch government,^8^ and a consortium of Nigerian scientists with support from the European Union and African Union. MRIGlobal operated the MDLs commissioned by Department of Defense in Guinea and Sierra Leone and tested thousands of samples safely.

There were numerous challenges and lessons learned while establishing the MDLs in Guinea and Sierra Leone. Many of the challenges were resolved by collaborating with the host government and other international partners. The primary goal of the MDLs was to provide a biologically safe laboratory to perform timely and quality diagnostics. However, without the dedicated support of engineering and logistics staff, the project would not have achieved a high level of success.

## Recommendations

The MRIGlobal MDLs in both Conakry and Freetown are still in use and will continue to be utilised by both countries, as well as international partners in the future. The diagnostic testing that is being performed in both laboratories has expanded over the past few years to include assays for influenza, severe acute respiratory syndrome coronavirus 2, Dengue, Chikungunya, Zika, and more. Using the MDLs for their molecular diagnostics is highly recommended until more sustainable solutions can be provided. Since their initial deployment, the MDLs in Sierra Leone and Guinea have increased both countries’ integrated disease surveillance and response systems, and adherence to international health regulations.^6^ In both Sierra Leone and Guinea, molecular testing for severe acute respiratory syndrome coronavirus 2 was performed using the capacity provided by the MDL. The MRIGlobal MDL provides a reproducible, strategic solution for the rapid deployment of molecular diagnostics in resource-limited settings. The strength of the MRIGlobal MDL is the ability to rapidly build and deploy it to almost anywhere in the world. However, the MRIGlobal MDL is expensive (other organisations deployed significantly cheaper laboratory operations that were of greater or equal sample testing efficiency and safety) and in resource-limited settings the MDLs are extremely challenging to maintain. Therefore, the deployment of MDLs should be carefully considered, given the cost and context.

## References

[1] Baize S, Pannetier D, Oestereich L, et al. Emergence of Zaire Ebola virus in Guinea. N Engl J Med. 2014;371:1418–1425. 10.1056/NEJMoa1404505 24738640

[2] World Health Organization. WHO Ebola situation report [homepage on the Internet]. 2016 [cited 2019 Mar 06]. Available from: http://apps.who.int/ebola/ebola-situation-reports

[3] Federal Select Agent Program. Select agents and toxins list [homepage on the internet]. 2017 [cited 2019 Mar 06]. Available from: https://www.selectagents.gov/SelectAgentsandToxinsList.html

[4] World Health Organization. Report on the status of EDPLN BSL-3 in select countries in the African Region, December 2016 [homepage on the Internet]. 2017 [cited 2019 Jun 12]. Available from: https://reliefweb.int/report/world/report-status-edpln-bsl-3-select-countries-african-region-december-2016

[5] 2014 Ebola Virus Emergency Use Authorizations. EZ1 Real-time RT-PCR Assay (DoD) – October 10, 2014 [homepage on the Internet]. 2014 [cited 2019 Jun 12]. Available from: https://www.fda.gov/media/89984/download

[6] World Health Organization. International Health Regulations 2005 Third Edition [homepage on the Internet]. 2016 [cited 2020 Dec 15]. Available from: https://www.who.int/publications/i/item/9789241580496

[7] Zhang Y, Yan G, Wang C, et al. Rapid deployment of a mobile biosafety level-3 laboratory in Sierra Leone during the 2014 Ebola virus epidemic. PLoS Negl Trop Dis. 2017;11(5):e0005622. 10.1371/journal.pntd.0005622 28505171 PMC5444861

[8] Reusken C, Smit P, Pas S, et al. Ebola virus laboratory response: The three Dutch Mobile laboratories in Liberia and Sierra Leone. Clin Microbiol Infect Dis. 2016;1(4):1–7. 10.15761/CMID.1000S1003

[9] Nigeria mobile lab in Sierra Leone: Bringing skills learned in one outbreak to another [homepage on the Internet]. 2015 [cited 2020 Aug 11]. Available from: https://www.afro.who.int/news/nigerian-mobile-lab-sierra-leone-bringing-skills-learned-one-outbreak-another

[10] Sealy TK, Erickson BR, Taboy CH, et al. Laboratory response to Ebola – West Africa and United States. MMWR Suppl. 2016;65(Suppl 3):44–49. 10.15585/mmwr.su6503a7 27389781

